# Supplementary material for: Novel Driver Strength Index highlights important cancer genes in TCGA PanCanAtlas patients
Source: PeerJ. 2022 Aug 11;10:e13860. doi: 10.7717/peerj.13860 (PMC9375969; doi:10.7717/peerj.13860)
Supplement: Supplemental Information 2 [file peerj-10-13860-s002.zip › COHORTS/genes plots/cohorts/2021_8_16_14_9_distribution_cohorts_males_CHOL.pdf]

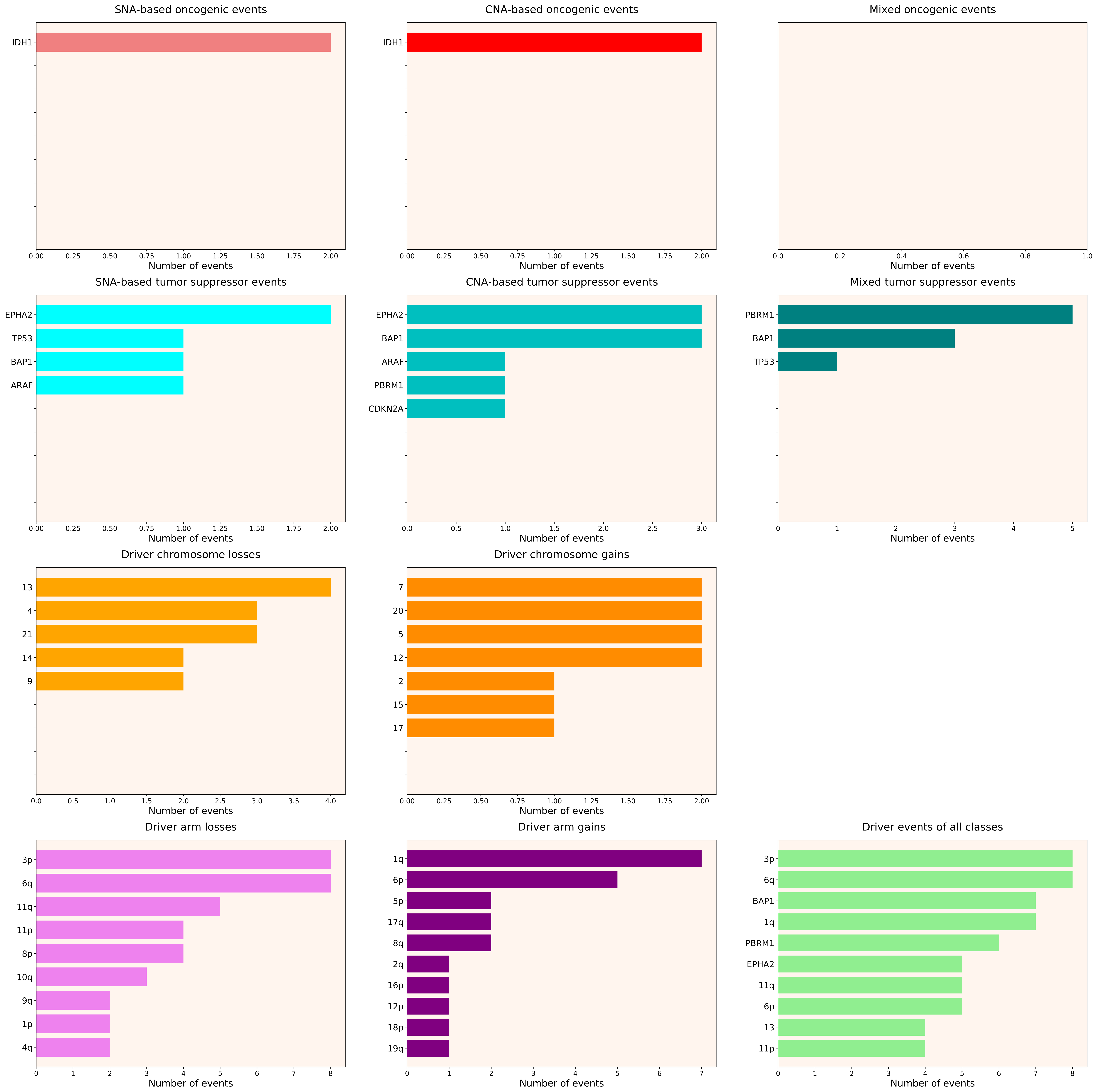
SNA-based oncogenic events
IDH1
Number of events
CNA-based oncogenic events
IDH1
Number of events
Mixed oncogenic events
Number of events
SNA-based tumor suppressor events
EPHA2
TP53
BAP1
ARAF
Number of events
CNA-based tumor suppressor events
EPHA2
BAP1
ARAF
PBRM1
CDKN2A
Number of events
Mixed tumor suppressor events
PBRM1
BAP1
TP53
Number of events
Driver chromosome losses
13
4
21
14
9
Number of events
Driver chromosome gains
7
20
5
12
2
15
17
Number of events
Driver arm losses
3p
6q
11q
11p
8p
10q
9q
1p
4q
Number of events
Driver arm gains
1q
6p
5p
17q
8q
2q
16p
12p
18p
19q
Number of events
Driver events of all classes
3p
6q
BAP1
1q
PBRM1
EPHA2
11q
6p
13
11p
Number of events
